# Diagnostic accuracy of tri-ponderal mass index and body mass index in estimating overweight and obesity in South African children

**DOI:** 10.4102/phcfm.v11i1.1949

**Published:** 2019-08-14

**Authors:** Violet K. Moselakgomo, Marlise van Staden

**Affiliations:** 1 Department of Physiology and Environmental Health, University of Limpopo, Sovenga, South Africa

**Keywords:** tri-ponderal mass index, body mass index, obesity, overweight, chronic diseases, South African children

## Abstract

**Background:**

Prevalence of obesity in youths has drastically increased in both industrialised and non-industrialised countries, and this transition resulted in an increased prevalence of chronic diseases.

**Aim:**

The study aimed to comparatively examine prevalence of overweight and obesity status based on tri-ponderal mass index and body mass index in estimating body fat levels in South African children.

**Setting:**

The study was conducted in Limpopo and Mpumalanga provinces of South Africa.

**Methods:**

A cross-sectional survey of 1361 (boys: *n* = 678; girls: *n* = 683) children aged 9–13 years was undertaken. The children’s age and sex-related measurements of body weight, waist-to-height ratio, waist-to-hip ratio, triceps skinfold, subscapular skinfolds and sum of skinfold were taken using the International Society for Advancement of Kinanthropometry protocol. TMI and BMI calculations classified children according to weight and age categories. Descriptive statistics, Spearman’s correlations and multiple linear regression analyses were set at ≤ 0.05.

**Results:**

Obesity classifications on TMI and BMI among children were as follows: Boys: 7.3%, 2.6%; 2.2%, 0.7%; Girls: 4.0%, 1.0%; 1.8%, 0.6%. Body weight, WHtR, WHpR, TSKF, SSKF and ΣSKF significantly correlated with TMI (*r* = 0.40, *p* < 0.001; *r* = 0.73, *p* < 0.001; *r* = −0.09, *p* < 0.001; *r* = 0.50, *p* < 0.001; *r* = 0.51, *p* < 0.001 and *r* = 0.52, *p* < 0.001) and BMI (*r* = 0.81, *p* < 0.001; *r* = 0.59, *p* < 0.001; *r* = −0.22, *p* < 0.001; *r* = 0.63, *p* < 0.001; *r* = 0.67, *p* < 0.001 and *r* = 0.66, *p* < 0.001). Regression analysis revealed that body weight, WHtR, WHpR, TSKF, SSKF and ΣSKF accounted for 65% and 85% of variance in children’s TMI (*R*^2^ = 0.647, *F*[6 1354] = 413.977, *p* < 0.001) and BMI (*R*^2^ = 0.851, *F*[6 1354] = 1288.218, *p* < 0.001).

**Conclusion:**

TMI revealed strikingly higher incidence of overweight and obesity in South African boys and girls than BMI. Future studies are needed to clarify sensitivity of TMI over BMI in quantifying obesity prevalence in children and adolescents.

## Introduction

The prevalence of obesity in youths has drastically increased in both industrialised and non-industrialised nations, thus threatening human health globally^1^; however, efforts are on-going to lower the menace of youthful excessive body fat.^2^ South Africa is a developing nation in sub-Saharan Africa, and the rate at which overweight and obesity continue to rise is of great concern. The current status of the epidemic prevalence in South African youth has doubled the global average.^3^ Therefore, the ever-rising incidence of overweight and obesity calls for urgent interventions through multi-disciplinary approaches.

The use of body mass index (BMI), calculated as weight in kilograms divided by height in meter squared, has been recognised as a useful tool for screening overweight and obesity globally; its outcome is based on adult body mass, being proportional to height squared.^2,4,5,6^ However, recently, its capacity to predict the percentage of fat mass has consistently been probed in the literature. It has been argued that during childhood and adolescent development, weight is not proportional to height squared.^2,4,5,6^ A BMI of over 30.0 kg/m^2^ has been established as the cut-off limit for the diagnosis of obesity in adults, thus weakening the legitimacy of BMI in youths. The contradiction in the mounting power of BMI then resulted in the use of BMI percentiles for each age (BMI z-score). Unfortunately, BMI z-score does not consider body compositions based on body size and body fat status transformation during adolescent growth.^2^ This discrepancy in BMI z-score then led to questioning the accuracy of BMI z-score in assessing obesity in youths which then calls for its replacement.^2^

Tri-ponderal mass index (TMI), weight divided by height cubed, has currently been reported and recommended for accuracy in assessing body fat levels in youths than BMI.^2^ Secondly, TMI has been regarded as a more reliable method for diagnosing overweight and obesity in large number of different ethnic groups, thus accounting for age and gender differences. Thirdly, it is sensitive in distinguishing transformation of fat mass distribution in over-fat children and variation in fat-free mass in lean children. It is specific and could predict both positive and negative values as compared with BMI. In addition, unlike BMI, TMI is recommended for its consistency in yielding accurate results in terms of body fat assessment for both tall and short individuals, thus maximising accuracy in screening for obesity and overweight status in children and adolescents. Given the cumulative prevalence of overweight and obesity in the South African population, it is worth exploring these indices for the estimation of obesity pandemic in South African youths. Therefore, the purpose of this study was to comparatively examine the prevalence of overweight and obesity based on the TMI and IOTF-BMI in South African children.

## Methods

### Study design

The study was conducted using a cross-sectional and quantitative descriptive design in which data were collected on body composition and anthropometric variables among school children and pre-adolescents based on a two-part relatively large-scale research of two under-resourced provinces of South Africa.

### Setting

The study was specifically conducted in the Limpopo and Mpumalanga provinces of South Africa. These provinces share a common boundary and are relatively under-resourced compared with other provinces in the country, and majority of the people belong to lower socio-economic brackets. Mostly, the population in these provinces earn their livelihood by subsistence farming or through work as migrant labourers. The Limpopo Province occupies an area of about 125 755 km^2^ with a population of about 5 404 868 people, mostly rural black people of the Pedi, Tsonga, Venda and other Bantu speaking people. Polokwane is its provincial capital. Mpumalanga, on the other hand, literally means the place where the sun rises. The province has only a surface area of 76 495 km^2^ with an estimated population of about 3 606 800 people comprising mainly of the Nguni including Swazi and Ndebele speaking people, and the white minority primarily speak Afrikaans in both the provinces. Mbombela is the provincial capital.

### Study population

From an estimated population of 15 000 learners, a total of 1361 children (*n* = 678 boys; *n* = 683 girls) aged 9–13 years were randomly selected from 8 schools in each of Limpopo (*n* = 708) and Mpumalanga (*n* = 653) rural settlements. Class registers were used to draw a targeted sample of 100 children from each school. Based on the total number of learners per class, serial numbers were written on small pieces of paper, folded and placed in an empty paper box. The pieces of paper were knotted and handpicked one after the other to select those who participate in the study. The participants’ ages were then verified from school register. Children who were outside of the age limits set for study were excluded from participation. Specifically, those who were present at the time of data collection and those aged 9–13 years were assessed.

### Sample size calculation

Slovin’s formula was used to determine the sample size of the study, which is as follows:

*n* = sample size of the adjusted population

*N* = population size

*e* = accepted level of error set at 0.05.

$$n=\frac{N}{1+N\times e^{2}}$$ [Eqn 1]

$$\begin{array}{l} n=\frac{15\;000}{1+15\;000\times {\left(0.05\right)}^{2}}\\ \;=15\;000/10.16=1476 \end{array}$$ [Eqn 2]

A total of 1476 children were selected to participate in the study; however, because of absenteeism and incomplete data on 115 participants, 1361 participants (678 boys and 689 girls) eventually completed the tests, and their data used in the final statistical analysis.

### Sampling strategy

A non-probability sampling was used in the study in which eight schools were purposively chosen from among other districts in the Limpopo and Mpumalanga provinces of South Africa. The rationale behind purposive sampling technique was that the schools were rural in nature and therefore included for the purpose of the study. Secondly, many schools in the area granted permission for data to be collected and study to be carried out. Thirdly, it was more feasible to conduct the research in rural settlements with the assistance of trained field workers, who were nursing and physical education students at the University of Limpopo, servicing both the provinces at the time, for proximity and accessibility purposes. In addition, it was more appropriate to collect data from two provinces because the schools were in rural settlements, and the pupil had a comparable socio-economic background.

### Data collection

#### Anthropometric measurements

Anthropometric measures including height, body weight, skinfolds (triceps and subscapular) and body girth (waist and hip) were evaluated according to the International Society for the Advancement of Kinanthropometry (ISAK) protocol.^7^ Height was measured to the nearest 0.1 cm in bare feet with participants standing upright against a stadiometer. A digital weighing scale (Tanita HD 309, Creative Products, MI, United States [US]) calibrated regularly to the nearest 0.1 kg (after every 10 measurements) measured body mass with participants lightly dressed (underwear and T-shirt). Based on the anthropometric measures, TMI (kg/height m^3^) and BMI (kg/height m^2^) were derived using the following equations:

$$\text{TMI}=\frac{\text{Body Mass }(\text{kg})}{{\left[\text{Height }(\text{m})\right]}^{3}}$$ [Eqn 3]

$$\text{BMI}=\frac{\text{Body Mass }(\text{kg})}{{\left[\text{Height }(\text{m})\right]}^{2}}$$ [Eqn 4]

Children’s anthropometric data were categorised as underweight, normal weight, overweight and/or obese for age and gender difference using TMI and BMI norms. For facilitation comparison, data of children who were severely light in weight were categorised as underweight, while data of those who were obese class I (moderately obese), obese class II (severely obese) and obese class III (very severely obese) were all collapsed and grouped as obese.

#### Circumferences

Waist girth was taken at the central point of the anatomical landmarks. Furthermore, readings were taken at the end of normal expiration with the arms naturally placed by the side.^7^ Hip (maximal girth of the buttocks) girth was measured thrice to the nearest 0.1 cm using the Gullick anthropometric tape while the participant assumed a standing position. The subject was asked to stand erect with feet together and weight evenly distributed. The widest part of the hip was located and marked, that is, at the level of greater trochanters. Based on data obtained, waist-to-height ratio (WHtR) was calculated as ratios of the children’s waist girth to their height, while waist-to-hip ratio (WHpR) was calculated as waist girth divided by hip girth, used to assess pattern of fat distribution among children.^8^

A pilot test was conducted for validation of the instrument and assessment procedures of which 20 children of the same age group who were not part of the main study participated. Pearson’s technique was used to determine the intra-observer reliability, technical error of measurement (TEM) as well as intra-class correlation coefficient (*r*) of anthropometric assessments^9^, and TEM was within satisfactory limits and agreed with suggestions of Lohman and Roche.^10^

### Data analysis

Anthropometric variables (body mass, BMI, TMI, WHtR, WHpR, triceps skinfold [TSKF], subscapular skinfolds [SSKF] and sum of skinfold [∑SKF]) were examined using independent samples *t*-test based on gender differences. Percentages for TMI and BMI were calculated for overweight and obesity classifications based on age and gender categories in the cohorts of South African children. Spearman’s *rho* correlation was performed to examine relationships between selected body composition variables, as well as TMI and BMI. Multiple linear regression analysis was similarly performed to predict TMI and BMI from selected body composition variables. All statistical analyses were conducted using the Statistical Package for Social Sciences (SPSS) version 25. For inferential data analyses, a probability level of ≤ 0.05 was taken to indicate significance.

### Ethical considerations

Ethics Sub-Committee of the Faculty of Health Sciences, North-West University, South Africa (Ethics no: NWU-00088-12-S1) and other relevant provincial regulatory bodies granted approval for the study to be carried out. Also, provincial heads of education department and district managers for Department of Basic Education in the Limpopo and Mpumalanga provinces gave permission for the study to be conducted. The research complied with the Health Professions Council of South Africa’s General Ethical Guidelines for Health Researchers^11^. All participants and their parents and guardians provided their written informed consent.

## Results

A significant difference in anthropometric and body composition characteristics between boys and girls is highlighted in Table 1. Participants’ mean age was 10.9 (1.30) years. At ages 9 and 10 years, girls had significantly higher WHtR (0.42; 0.43) and WHpR (0.85; 0.83) than boys whose corresponding mean values were 0.41; 0.42 and 0.81; 0.79, respectively (*p* > 0.05). Boys had significantly higher mean values of triceps (9.24 mm, 10.4 mm, 10.9 mm, 11.8 mm and 13.4 mm), subscapular (5.50 mm, 6.30 mm, 6.91 mm, 7.93 mm and 9.47 mm) and sum of skinfolds (ΣSKF) (14.7 mm, 16.8 mm, 17.8 mm, 19.7 mm and 22.9 mm) than girls (*p* > 0.05). Also at ages 12 and 13 years, boys were significantly heavier (39.6 kg; 46.1 kg) and had higher BMI (18.6 kg/m^2^; 20.0 kg/m^2^) and TMI (12.8 kg/m^3^; 13.3 kg/m^3^) scores compared to their girl counterparts whose corresponding mean values were body mass: 35.3 kg, 40.3 kg; BMI: 17.3 kg/m^2^, 18.0 kg/m^2^; and TMI: 12.2 kg/m^3^, 12.1 kg/m^3^, respectively (*p* > 0.05). No significant mean values were observed for body mass, BMI and TMI at ages 9, 10 and 11 years for boys and girls, respectively (*p* > 0.05), although body mass (27.5 kg, 31.5 kg, 34.9 kg, 39.6 kg and 46.1 kg), BMI (15.9 kg/m^2^, 17.1 kg/m^2^, 17.5 kg/m^2^, 18.6 kg/m^2^ and 20.0 kg/m^2^), TSKF (9.24 mm, 10.4 mm, 10.9 mm, 11.8 mm and 13.4 mm) SSKF (5.50 mm, 6.30 mm, 6.91 mm, 7.93 mm and 9.47 mm) and ∑SKF (14.7 mm, 16.8 mm, 17.8 mm, 19.7 mm and 22.9 mm) consistently increased with age among the boys, in contrast to the girls. However, body mass (27.4 kg, 31.1 kg, 33.1 kg, 35.3 kg and 40.3 kg) was found to have consistently increased with age among the girls.

**TABLE 1:** Anthropometric characteristics of South Africa children by sex and age categories.

Gender	Age	Sample size	Body mass (kg)		BMI (kg/m^2^)		TMI (kg/m^3^)		WHtR		WHpR		TSKF (mm)		SSKF (mm)		ΣSKF	
			M	s.d.	M	s.d.	M	s.d.	M	s.d.	M	s.d.	M	s.d.	M	s.d.	M	s.d.
Boys	9	113	27.5	5.42	15.9	1.95	12.2	1.41	0.41*	0.03	0.81*	0.04	9.24*	3.54	5.50*	3.05	14.7*	6.38
	10	170	31.5	6.36	17.1	2.35	12.6	1.63	0.42*	0.04	0.79*	0.04	10.4*	4.15	6.30*	3.01	16.8*	7.14
	11	172	34.9	8.17	17.5	3.13	12.5	2.11	0.41	0.04	0.79*	0.04	10.9*	4.38	6.91*	3.73	17.8*	7.90
	12	144	39.6*	9.37	18.6*	4.41	12.8*	3.83	0.42	0.05	0.77*	0.05	11.8*	5.08	7.93*	4.21	19.7*	9.11
	13	84	46.1*	9.65	20.0*	3.84	13.3*	3.08	0.43*	0.05	0.77*	0.05	13.4*	5.50	9.47*	4.31	22.9*	9.59
Girls	9	95	27.4	4.79	16	1.75	12.3	1.29	0.42*	0.03	0.85*	0.04	7.54*	3.46	4.50*	3.48	12.1*	6.74
	10	145	31.1	6.25	17.2	2.98	12.7	2.29	0.43*	0.04	0.83*	0.04	8.57*	3.51	5.20*	2.66	13.7*	6.03
	11	171	33.1	10.3	17.3	4.72	12.5	3.30	0.42	0.04	0.83*	0.04	8.12*	3.25	4.90*	2.58	13.0*	5.64
	12	153	35.3*	7.71	17.3*	2.59	12.2*	1.71	0.42	0.03	0.83*	0.05	8.43*	3.61	5.30*	2.95	13.7*	6.43
	13	114	40.3*	7.32	18.0*	2.21	12.1*	1.49	0.42*	0.03	0.82*	0.05	9.76*	4.70	6.02*	3.17	15.8*	7.67

kg, kilogram; BMI, body mass index; kg/m^2^, kilogram per meter square; TMI, tri-ponderal index; kg/m^3^, kilogram per meter cubic; WHtR, waist-to-height ratio; WHpR, waist-to-hip ratio; TSKF, triceps skinfold; mm, millimetre; SSKF, subscapular skinfold; ΣSKF, sum of skinfolds; M, mean; s.d., standard deviation.

* , Statistically significant (*p* ≤ 0.05).

Percentage of BMI and TMI classifications as stratified by gender indicated that 7.3% (TMI ≥ 15) boys were more overweight than 2.6% (BMI ≥ 25) fellows. With regard to girls, 4.0% (TMI ≥ 15) exhibited an increased level of overweight compared to 1.0% (BMI ≥ 25) girls. The children’s TMI scores indicated a higher prevalence of obesity among the boys (2.2%) and girls (1.8%) (TMI ≥ 17.5) than their BMI classification whose data were as follows (Table 2): boys (0.7%) and girls (0.6%).

**TABLE 2:** Percentage of body mass index and tri-ponderal mass index classifications of South African children by gender.

Index categories	Body mass index				Tri-ponderal mass index			
	Boys		Girls		Boys		Girls	
	No.	%	No.	%	No.	%	No.	%
Underweight	471	69.0	544	80.2	128	18.7	114	16.8
Normal weight	189	27.7	123	18.1	490	71.7	525	77.4
Overweight	18	2.6	7	1.0	50	7.3	27	4.0
Obese	5	0.7	4	0.6	15	2.2	12	1.8

**Total**	**683**	**100**	**678**	**100**	**683**	**100**	**678**	**100**

*p* < 0.05.

Table 3 presents the BMI and TMI overweight and obesity classifications of the children. A significant difference between BMI and TMI categories in the children was observed. The results indicated 1.8% (BMI) and 5.7% (TMI) for overweight categories, while 0.7% and 2.0% data were found for obesity categories.

**TABLE 3:** Diagnostic classification of obesity among South African children based on body mass index (IOTF) and tri-ponderal mass Index category by age.

Index categories	Age											
	9 years		10 years		11 years		12 years		13 years		Total	
	N	%	N	%	N	%	N	%	N	%	N	%
**BMI (IOTF)**												
Underweight	186	89.4	249	79.0	268	78.1	205	69.0	107	54.0	1015	74.6
Normal weight	22	10.6	62	19.7	67	19.5	79	26.6	82	41.4	312	22.9
Overweight	0	0.0	3	1.0	5	1.5	9	3.0	8	4.0	25	1.8
Obese	0	0.0	1	0.3	3	0.9	4	1.3	1	0.5	9	0.7
**TMI**												
Underweight	33	15.9	43	13.7	61	17.8	68	22.9	37	18.7	242	17.8
Normal weight	167	80.3	245	77.8	258	75.2	207	69.7	138)	69.7	1015	74.6
Overweight	8	3.8	20	6.3	17	5.0	12	4.0	20	10.1	77	5.7
Obese	0	0.0	7	2.2	7	2.0	10	3.4	3	1.5	27	2.0

IOTF-BMI, International obesity task force-body mass index; TMI, tri-ponderal mass index.

Shown in Table 4 is the Spearman’s rho bivariate correlation between TMI, BMI and selected body composition variables. Tri-ponderal mass index significantly correlated positively with body mass (*r* = 0.40; *p* < 0.001), WHtR (*r* = 0.73; *p* < 0.001), TSKF (*r* = 0.50; *p* < 0.001), SSKF (*r* = 0.51; *p* < 0.001) and ΣSKF (*r* = 0.52; *p* < 0.001), but inversely correlated with WHpR (*r* = −0.09; *p* < 0.001). On the other hand, body mass index significantly correlated with body mass (*r* = 0.81; *p* < 0.001), WHtR (*r* = 0.60; *p* < 0.001), TSKF (*r* = 0.63; *p* < 0.001), SSKF (*r* = 0.67; *p* < 0.001) and ΣSKF (*r* = 0.66; *p* < 0.001), but significantly inversely correlated with WHpR (*r* = −0.22; *p* < 0.001).

**TABLE 4:** Spearman’s *rho* correlation matrix showing interrelationships between selected body composition variables and tri-ponderal mass index and body mass index in estimating body fat levels in South African children (*n* = 1361).

Variables	Weight (kg)	WHtR	WHpR	TSKF (mm)	SSKF (mm)	ΣSKF	BMI (kg/m^2^)	TMI (kg/m^3^)
Weight (kg)	1.000	0.280**	−0.292**	0.567**	0.634**	0.609**	0.813**	0.402**
WHtR	0.280**	1.000	0.372**	0.336**	0.380**	0.360**	0.598**	0.727**
WHpR	−0.292**	0.372**	1.000	−0.338**	−0.318**	−0.341**	−0.222**	−0.090**
TSKF (mm)	0.567**	0.336**	−0.338**	1.000	0.870**	0.979**	0.627**	0.500**
SSKF (mm)	0.634**	0.380**	−0.318**	0.870**	1.000	0.949**	0.673**	0.514**
ΣSKF	0.609**	0.360**	−0.341**	0.979**	0.949**	1.000	0.662**	0.519**
BMI (kg/m^2^)	0.813**	0.598**	−0.222**	0.627**	0.673**	0.662**	1.000	0.840**
TMI (kg/m^3^)	0.402**	0.727**	−0.090**	0.500**	0.514**	0.519**	0.840**	1.000

BMI, body mass index; kg, kilogram; ΣSKF, sum of skinfolds; SSKF, subscapular skinfolds; TMI, tri-ponderal mass index; TSKF, triceps skinfold; WHpR, waist-to-hip ratio; WHtR, waist-to-height ratio.

** , Correlation is significant at the 0.01 level (two-tailed).

Results of the multiple linear regression analysis performed to predict TMI and BMI based on participants’ body weight, WHtR, WHpR, TSKF, SSKF and ΣSKF are provided in Table 5. The results revealed that every kilogram change in body mass could lead to a higher significant increase in both TMI and BMI by 0.06 kg and 0.22 kg, respectively. Also, every unit change in WHtR could lead to a significant increase in TMI and BMI by 46.7 and 42.5, respectively. Every unit increase in WHpR could translate to a higher significant decrease in TMI and BMI by 14.4 and 13.0, respectively. Furthermore, every millimetre change in TSKF could insignificantly lead to an increase in both BMI and TMI by 0.05 mm and 0.04 mm, respectively. Results also showed that every millimetre change in subscapular skinfold could yield a non-significant decrease in both BMI and TMI by 0.10 mm and 0.10 mm, respectively, while every unit change in ΣSKF could lead to non-significant decrease in both BMI and TMI by 0.03 mm and 0.01 mm, respectively.

**TABLE 5:** Multiple linear regression analysis to predict tri-ponderal mass index and body mass index from selected body composition variables.

Variables	Tri-ponderal index† – Coefficient‡	*p*	Body mass index† – Coefficient†	*p*
Weight (kg)	0.060	< 0.001	0.223	< 0.001
WHtR	46.741	< 0.001	42.552	< 0.001
WHpR	−14.421	< 0.001	−13.022	< 0.001
TSKF (mm)	0.054	0.493	0.038	0.587
SSKF (mm)	−0.089	0.275	−0.094	0.201
ΣSKF	−0.031	0.684	−0.014	0.833

ΣSKF, sum of skinfolds; SSKF, subscapular skinfolds; TSKF, triceps skinfold; WHpR, waist-to-hip ratio; WHtR, waist-to-height ratio.

† , *R*^2^ =0.647; adjusted *R*^2^ =0.646; *p* < 0.05.

‡ , *R*^2^ =0.851; adjusted *R*^2^ = 0.850; *p* < 0.05.

A generalised equation obtained from coefficients presented in Table 5 was used to predict TMI from body weight, WHtR, WHpR, TSKF, SSKF and ΣSKF as follows: TMI = 0.060 (weight) + 46.741 (WHtR) + 0.054 (TSKF) − 14.421 (WHpR) − 0.089 (SSKF) – 0.031 (ΣSKF). The variable significantly predicted TMI, *F* (6 1354) = 413.977, *p* < 0.001, *R*^2^ = 0.647; *p* < 0.05. Therefore, only body weight, WHtR and WHpR were significant predictors of TMI among the children. Conversely, the generalised equation used to predict BMI from body weight, WHtR, WHpR, TSKF, SSKF and ΣSKF was: BMI = 0.223 (weight) + 42.552 (WHtR) + 0.038 (TSKF) − 13.022 (WHpR) − 0.094 (SSKF) – 0.014 (ΣSKF), as indicated in Table 5. The variables significantly predicted BMI, *F* (6 1354) = 1288.218, *p* < 0.001, *R*^2^ = 0.851; *p* < 0.05, thereby suggesting that body weight, WHtR and WHpR were the most significant predictors of BMI among the South African children.

## Discussion

This study comparatively examined the prevalence of overweight and obesity in South African children based on tri-ponderal and body mass indices. Body mass index compared with TMI has been commonly used as a tool to screen for obesity prevalence in adults and used by many researchers to assess body fatness and body weight disorders in children because of its relative simplicity and ease of determination. However, studies have shown that BMI may not have the same proportion with height squared in its use to assess body fat levels in children,^2^ thus limiting its sensitivity for evaluating fatness in children and adolescents. Historically, BMI was an ideal and preferred body weight index measure for diverse population across the globe, while ponderal index was considered a poor approach.^12^ Blackburn and Jacobs^13^ were of the opinion that BMI was considered the best measure of fatness when several issues were not put into consideration. For example, (1) children, women and the old were not investigated; and (2) changes in BMI with visceral fat patterns were not observed.^10,14^ These shortcomings coupled with BMI’s inability to separate body fat from fat-free mass tend to weaken the precision of the index as a measure of fatness and heaviness.

In our study sample, comparison of the tri-ponderal and body mass indices yielded different results. There was a higher prevalence of overweight and obesity using the TMI compared to the BMI in both boys and girls. The metamorphosis perceived in both boys and girls in this study rationalises the prerequisite to reflect age- and gender-specific inceptions as such thresholds are deliberated in BMI evaluations for children and adolescents, given the cumulative prevalence of overweight and obesity in the South African population. However, it contradicts that of an Indian study in which a lower prevalence of overweight and obesity^15^ was found when BMI-IOTF was used to estimate pandemic in children aged 2–17 years category. Khadilkar and colleagues,^15^ however, coincide with those studies conducted in Nigeria^16^ in which a higher prevalence of overweight and obesity was found for Centre for Disease Control and Prevention estimation compared with the BMI-IOTF. These findings suggest higher responsiveness and stringency in BMI-IOTF compared to both Centre for Disease Control and Prevention and TMI in youths. On the other hand, such rigours might have resulted in an underestimation of overweight and obesity in youths. Alternatively, variances in ethnicity or population, genetic predisposition, lifestyle regime and age range as epidemic determinant might denote that the data are not solidly comparable.

A strong positive correlation was observed between BMI and body weight in this study, in contrast to the significantly moderate correlation noted for TMI. The finding also corroborates that of Howe and colleagues,^1^ which indicated higher significant association between TMI and BMI and greater fat weight among youths. Howe and colleagues^1^ stated further that an increase in BMI could be linked to various cardio-metabolic disease risks among youths. Similar to this study, Rasmussen and Johansson^17^ also found significantly positive relationship between TMI and body mass in their study which analysed the importance of birth mass, height and Rohrer index for BMI and overweight or obesity at 18 years of age. These findings have significant clinical repercussions and thus advocate for premature acknowledgement of amplified cardio-metabolic disease risks at young ages. Dancey and Reidy’s^18^ correlation interpretation also supported the findings by Howe and colleagues^1^ as having an increased BMI could be interrelated with numerous cardio-metabolic disease risks among youths and future negative health impact later in life.

The results of this study also indicated a significantly higher correlation with regard to WHtR and TMI, whereas triceps, subscapular and summation of skinfold significantly moderately correlated with both TMI and BMI. These findings corroborate those of Sanya and Alaje^19^ which observed a considerable correlation of skinfolds with both BMI and TMI between gender and among different age groups. In another study, Gaskin and Walker^20^ examined the relationships of BMI to obesity indices tracking excessive fat between late childhood and early adolescence in a cohort of children with various nutritional statuses and found a strong relationship between obesity indices and BMI. Similar to this study, WHpR had a low inverse significant correlation with both TMI and BMI, although higher with BMI.

Findings from the regression analysis revealed that only three independent variables (body weight, WHtR and WHpR) highly significantly predicted TMI and BMI in the studied population. Our findings substantiate those studies in which body mass significantly predicted BMI.^16^ Other studies also found BMI and TMI to be associated with cardiovascular diseases among individuals,^2,21,22,23,24^ despite the criticism of TMI being regarded as poor relative weight index.^23,25^

The fact that other studies reported lack of consistency in standard body fat classification when evaluating weight abnormalities in youths suggests that the assessment of overweight and obesity in children should be based on good results as this could weaken the reliability of epidemiological research data. The results of this study therefore advocate a requisite for an evaluative approach to institute standards for the assessment of percentage body in children and associate the outcomes on the basis of age and gender. In addition, it will be ideal to explore a diverse of indices for the determination of body fat in South African children.

The present findings should, however, be interpreted cautiously in view of the following limitations: Firstly, the BMI and TMI might not represent the right body proportions which could incorporate both fat mass and fat-free mass, thus limiting their accuracy to estimate obesity and overweight in children. Secondly, participants in this study were from low-resourced communities, and therefore, the findings are not representative of the population of South African children.

## Conclusion

Tri-ponderal mass index revealed strikingly higher prevalence of overweight and obesity in the South African boys and girls compared with the BMI. Future studies are necessary to clarify the sensitivity of the TMI over the IOTF-based BMI classifications in estimating body fatness in children and adolescents; as such findings could have implications for public health policy formulation.
